# Supplementary material for: A systematic review of behaviour change interventions to improve maternal health outcomes in sub-Saharan Africa
Source: PLOS Glob Public Health. 2024 Feb 20;4(2):e0002950. doi: 10.1371/journal.pgph.0002950 (PMC10878526; doi:10.1371/journal.pgph.0002950)
Supplement: S1 Table — (DOCX) [file pgph.0002950.s004.docx]

A systematic review of behavioural approaches for improving maternal health outcomes in Africa.

**Search strategy**
**Databases: PsycINFO, Econlit, Cochrane Database of Systematic reviews, International Bibliography of Social Sciences, EMBASE, MEDLINE, CINAHL PLUS,**  SCOPUS, African Index Medicus, African Journals Online (AJOL), and Web of Science.
**Hand searches – Annals of Behavioural Medicine, Health Psychology, Implementation Science, Social Science and Medicine**.

| **Descriptors** | **Population (Patients, Partners, Health Workers, Families, Communities, Managers etc)** | **+** | **Concept TiAb (Behaviour/Behaviour Change Interventions)** | **+** | **Concept TiAb (Behaviour change implementation strategy** | **+** | **Context TiAb** | **Study design** |
| --- | --- | --- | --- | --- | --- | --- | --- | --- |
| **Keywords/synonyms** | **"Pregnancy"[MeSH Terms] OR "Gravidity"[MeSH Terms] OR "Pregnant Women"[MeSH Terms] OR "Prenatal Care"[MeSH Terms] OR "Perinatal Care"[MeSH Terms] OR "Prenatal Diagnosis"[MeSH Terms] OR "Obstetrics"[MeSH Terms] OR "pregnan*"[Title/Abstract] OR "gravid*"[Title/Abstract] OR "gestat*"[Title/Abstract] OR "pregnant Women"[Title/Abstract] OR "pregnant woman"[Title/Abstract] OR "periconception"[Title/Abstract] OR "childbear*"[Title/Abstract] OR "childbirth"[Title/Abstract] OR "birth*"[Title/Abstract] OR "deliver*"[Title/Abstract] OR "parturition"[Title/Abstract] OR "maternity"[Title/Abstract] OR "matern*"[Title/Abstract] "obstetric*"[Title/Abstract] OR "prepartum"[Title/Abstract] OR "antepartum"[Title/Abstract] OR "prenat*"[Title/Abstract] OR "antenat*"[Title/Abstract] OR "perinat*"[Title/Abstract] OR "intrapartum"[Title/Abstract] OR "labour"[Title/Abstract] OR "labor"[Title/Abstract] OR "perinat*"[Title/Abstract] OR "postnat*"[Title/Abstract] OR "postpregnancy"[Title/Abstract] OR "after pregnancy"[Title/Abstract] OR "postpartum"[Title/Abstract] OR "partner*"[Title/Abstract] OR "husband*"[Title/Abstract] OR "birth partner"[Title/Abstract] OR "obstetrician*"[Title/Abstract] OR "midwife*"[Title/Abstract] OR "midwive*"[Title/Abstract] OR "nurse midwife*"[Title/Abstract] OR "nurse midwive*"[Title/Abstract] OR "health worker*"[Title/Abstract] OR "healthworker*"[Title/Abstract] OR "health care workers"[Title/Abstract] OR "manager*"[Title/Abstract] OR "policy maker*"[Title/Abstract] OR "leader*"[Title/Abstract] OR “famil*”[Title/Abstract] OR “communit*”[Title/Abstract]** |  | **"behaviour*"[Title/Abstract] OR "behavior*"[Title/Abstract] OR "behaviour change"[Title/Abstract] OR "behavior change*"[Title/Abstract] OR "behaviour change intervention*"[Title/Abstract] OR "behaviour change program*"[Title/Abstract] OR "behaviour change programme*"[Title/Abstract] OR "behaviour change techniques*"[Title/Abstract] OR "behaviour change strategy*"[Title/Abstract] OR "behaviour change strategies*"[Title/Abstract] OR "behaviour change approach*"[Title/Abstract] OR "behavior change intervention*"[Title/Abstract] OR "behavior change program*"[Title/Abstract] OR "behavior change techniques*"[Title/Abstract] OR "behavior change strategy*"[Title/Abstract] OR "behavior change strategies*"[Title/Abstract] OR "behavior change approach*"[Title/Abstract] OR "attitude*"[Title/Abstract] OR "attitude change*"[Title/Abstract] OR "attitude change intervention*"[Title/Abstract] OR "attitude change program*"[Title/Abstract] OR "attitude change programme*"[Title/Abstract] OR "attitude change techniques*"[Title/Abstract] OR "attitude change strateg*"[Title/Abstract] OR "attitude change strategies*"[Title/Abstract] OR "attitude approach*"[Title/Abstract] OR "conduct*"[Title/Abstract] OR "conduct intervention*"[Title/Abstract] OR "conduct program*"[Title/Abstract] OR "conduct strateg*"[Title/Abstract] OR "demeanour"[Title/Abstract] OR "character"[Title/Abstract] OR "character change*"[Title/Abstract] OR "character approach*"[Title/Abstract] OR "manners*"[Title/Abstract] OR "habits"[Title/Abstract] OR "habits change*"[Title/Abstract] OR "etiquette"[Title/Abstract] OR "etiquette strategy*"[Title/Abstract] OR "respectful care*"[Title/Abstract]** |  | **“educat*”[Title/Abstract] OR “persua*”[Title/Abstract] OR “incentiv*”[Title/Abstract] OR “reward*”[Title/Abstract] OR “prize*”[Title/Abstract] OR “present*”[Title/Abstract] OR “award*”[Title/Abstract] OR “recogni*”[Title/Abstract] OR “coerc*”[Title/Abstract] OR “train*”[Title/Abstract] OR “enable*”[Title/Abstract] OR “support*”[Title/Abstract] OR “model*”[Title/Abstract] OR “demonstrat*”[Title/Abstract] OR “showcas*”[Title/Abstract] OR “practic*”[Title/Abstract] OR “rehears*”[Title/Abstract] OR “environmental restructuring” [Title/Abstract] OR “restrict*”[Title/Abstract] OR “audit” [Title/Abstract] OR “feedback” [Title/Abstract] OR “clinical incident reporting” [Title/Abstract] OR “performance monitoring”[Title/Abstract] OR “communities of practice” [Title/Abstract] OR “continuous quality improvement” [Title/Abstract] OR “educational games” [Title/Abstract] OR “educational materials” [Title/Abstract] OR “educational meetings” [Title/Abstract] OR “educational outreach visits” [Title/Abstract] OR “academic detailing” [Title/Abstract] OR “clinical practice guidelines"[Title/Abstract] OR “interprofessional education” [Title/Abstract] OR “consensus” [Title/Abstract] OR “opinion leader*”[Title/Abstract] OR “champion”[Title/Abstract] OR “managerial supervision” [Title/Abstract] OR “patient mediated interventions” [Title/Abstract] OR “public release”[Title/Abstract] OR “performance data”[Title/Abstract] OR “reminders” [Title/Abstract] OR “patient reported outcome measure*”[Title/Abstract] OR “tailored interventions” [Title/Abstract]** |  | **"africa*"[Title/Abstract] OR "Sub-Saharan Africa"[Title/Abstract] OR "Algeria"[Title/Abstract] OR "Angola"[Title/Abstract] OR "Benin"[Title/Abstract] OR "Botswana"[Title/Abstract] OR "Burkina Faso"[Title/Abstract] OR "Burundi"[Title/Abstract] OR "Cape Verde"[Title/Abstract] OR "Cabo Verde"[Title/Abstract] OR "Cameroon"[Title/Abstract] OR "Central African Republic"[Title/Abstract] OR "Chad"[Title/Abstract] OR "Comoros"[Title/Abstract] OR "Democratic Republic of the Congo"[Title/Abstract] OR "Republic of Congo"[Title/Abstract] OR "Cote d'Ivoire"[Title/Abstract] OR "Ivory Coast"[Title/Abstract] OR "Djibouti"[Title/Abstract] OR "Egypt"[Title/Abstract] OR "Equatorial Guinea"[Title/Abstract] OR "Eritrea"[Title/Abstract] OR "Swaziland"[Title/Abstract] OR "Eswatini"[Title/Abstract] OR "Ethiopia"[Title/Abstract] OR "Gabon"[Title/Abstract] OR "Gambia"[Title/Abstract] OR "Ghana"[Title/Abstract] OR "Guinea"[Title/Abstract] OR "Guinea-Bissau"[Title/Abstract] OR "Kenya"[Title/Abstract] OR "Lesotho"[Title/Abstract] OR "Liberia"[Title/Abstract] OR "Libya"[Title/Abstract] OR "Madagascar"[Title/Abstract] OR "Malawi"[Title/Abstract] OR "Mali"[Title/Abstract] OR "Mauritania"[Title/Abstract] OR "Mauritius"[Title/Abstract] OR "Morocco"[Title/Abstract] OR "Mozambique"[Title/Abstract] OR "Namibia"[Title/Abstract] OR "Niger"[Title/Abstract] OR "Nigeria"[Title/Abstract] OR "Rwanda"[Title/Abstract] OR "Sao Tome and Principe"[Title/Abstract] OR "Senegal"[Title/Abstract] OR "Seychelles"[Title/Abstract] OR "Sierra Leone"[Title/Abstract] OR "Somalia"[Title/Abstract] OR "South Africa"[Title/Abstract] OR "South Sudan"[Title/Abstract] OR "Sudan"[Title/Abstract] OR "Tanzania"[Title/Abstract] OR "Togo"[Title/Abstract] OR "Tunisia"[Title/Abstract] OR "Uganda"[Title/Abstract] OR "Zambia"[Title/Abstract] OR "Zimbabwe"[Title/Abstract]** | **"trial*"[Title/Abstract] OR "quasi* experimental*"[Title/Abstract] OR "quasi*"[Title/Abstract] OR "quasi - experimental*"[Title/Abstract] OR "non randomized"[Title/Abstract] OR "non randomised"[Title/Abstract] OR "experimental"[Title/Abstract] OR "before* and after*"[Title/Abstract] OR "pre* and post*"[Title/Abstract] OR "pretest and posttest"[Title/Abstract] OR "non equivalent"[Title/Abstract] OR "combination* design*"[Title/Abstract] OR "implementation*"[Title/Abstract] OR "interrupted time series*"[Title/Abstract] OR "Natural experiment*"[Title/Abstract] OR "pseudo experiment*"[Title/Abstract] OR "feasibility"[Title/Abstract]** |
|  |  |  |  |  |  |  |  |  |

**Updated search strategy output on PubMed**(((("Pregnancy"[MeSH Terms] OR "Gravidity"[MeSH Terms] OR "pregnant women"[MeSH Terms] OR "Prenatal Care"[MeSH Terms] OR "Perinatal Care"[MeSH Terms] OR "Prenatal Diagnosis"[MeSH Terms] OR "Obstetrics"[MeSH Terms] OR "pregnan*"[Title/Abstract] OR "gravid*"[Title/Abstract] OR "gestat*"[Title/Abstract] OR "pregnant women"[Title/Abstract] OR "pregnant woman"[Title/Abstract] OR "periconception"[Title/Abstract] OR "childbear*"[Title/Abstract] OR "childbirth"[Title/Abstract] OR "birth*"[Title/Abstract] OR "deliver*"[Title/Abstract] OR "parturition"[Title/Abstract] OR "maternity"[Title/Abstract] OR "matern*"[Title/Abstract]) AND "obstetric*"[Title/Abstract]) OR "prepartum"[Title/Abstract] OR "antepartum"[Title/Abstract] OR "prenat*"[Title/Abstract] OR "antenat*"[Title/Abstract] OR "perinat*"[Title/Abstract] OR "intrapartum"[Title/Abstract] OR "labour"[Title/Abstract] OR "labor"[Title/Abstract] OR "perinat*"[Title/Abstract] OR "postnat*"[Title/Abstract] OR "postpregnancy"[Title/Abstract] OR "after pregnancy"[Title/Abstract] OR "postpartum"[Title/Abstract] OR "partner*"[Title/Abstract] OR "husband*"[Title/Abstract] OR "birth partner"[Title/Abstract] OR "obstetrician*"[Title/Abstract] OR "midwife*"[Title/Abstract] OR "midwive*"[Title/Abstract] OR "nurse midwife*"[Title/Abstract] OR "nurse midwive*"[Title/Abstract] OR "health worker*"[Title/Abstract] OR "healthworker*"[Title/Abstract] OR "health care workers"[Title/Abstract] OR "manager*"[Title/Abstract] OR "policy maker*"[Title/Abstract] OR "leader*"[Title/Abstract] OR "famil*"[Title/Abstract] OR "communit*"[Title/Abstract]) AND ("behaviour*"[Title/Abstract] OR "behavior*"[Title/Abstract] OR "behaviour change"[Title/Abstract] OR "behavior change*"[Title/Abstract] OR "behaviour change intervention*"[Title/Abstract] OR "behaviour change program*"[Title/Abstract] OR "behaviour change programme*"[Title/Abstract] OR "behaviour change techniques*"[Title/Abstract] OR "behaviour change strategy*"[Title/Abstract] OR "behaviour change strategies*"[Title/Abstract] OR "behaviour change approach*"[Title/Abstract] OR "behavior change intervention*"[Title/Abstract] OR "behavior change program*"[Title/Abstract] OR "behavior change techniques*"[Title/Abstract] OR "behavior change strategy*"[Title/Abstract] OR "behavior change strategies*"[Title/Abstract] OR "behavior change approach*"[Title/Abstract] OR "attitude*"[Title/Abstract] OR "attitude change*"[Title/Abstract] OR "attitude change intervention*"[Title/Abstract] OR "attitude change program*"[Title/Abstract] OR "attitude change programme*"[Title/Abstract] OR "attitude change techniques*"[Title/Abstract] OR "attitude change strateg*"[Title/Abstract] OR "attitude change strategies*"[Title/Abstract] OR "attitude approach*"[Title/Abstract] OR "conduct*"[Title/Abstract] OR "conduct intervention*"[Title/Abstract] OR "conduct program*"[Title/Abstract] OR "conduct strateg*"[Title/Abstract] OR "demeanour"[Title/Abstract] OR "character"[Title/Abstract] OR "character change*"[Title/Abstract] OR "character approach*"[Title/Abstract] OR "manners*"[Title/Abstract] OR "habits"[Title/Abstract] OR "habits change*"[Title/Abstract] OR "etiquette"[Title/Abstract] OR "etiquette strategy*"[Title/Abstract] OR "respectful care*"[Title/Abstract]) AND ("educat*"[Title/Abstract] OR "persua*"[Title/Abstract] OR "incentiv*"[Title/Abstract] OR "reward*"[Title/Abstract] OR "prize*"[Title/Abstract] OR "present*"[Title/Abstract] OR "award*"[Title/Abstract] OR "recogni*"[Title/Abstract] OR "coerc*"[Title/Abstract] OR "train*"[Title/Abstract] OR "enable*"[Title/Abstract] OR "support*"[Title/Abstract] OR "model*"[Title/Abstract] OR "demonstrat*"[Title/Abstract] OR "showcas*"[Title/Abstract] OR "practic*"[Title/Abstract] OR "rehears*"[Title/Abstract] OR "environmental restructuring"[Title/Abstract] OR "restrict*"[Title/Abstract] OR "audit"[Title/Abstract] OR "feedback"[Title/Abstract] OR "clinical incident reporting"[Title/Abstract] OR "performance monitoring"[Title/Abstract] OR "communities of practice"[Title/Abstract] OR "continuous quality improvement"[Title/Abstract] OR "educational games"[Title/Abstract] OR "educational materials"[Title/Abstract] OR "educational meetings"[Title/Abstract] OR "educational outreach visits"[Title/Abstract] OR "academic detailing"[Title/Abstract] OR "clinical practice guidelines"[Title/Abstract] OR "interprofessional education"[Title/Abstract] OR "consensus"[Title/Abstract] OR "opinion leader*"[Title/Abstract] OR "champion"[Title/Abstract] OR "managerial supervision"[Title/Abstract] OR "patient mediated interventions"[Title/Abstract] OR "public release"[Title/Abstract] OR "performance data"[Title/Abstract] OR "reminders"[Title/Abstract] OR "patient reported outcome measure*"[Title/Abstract] OR "tailored interventions"[Title/Abstract]) AND ("africa*"[Title/Abstract] OR "Sub-Saharan Africa"[Title/Abstract] OR "Algeria"[Title/Abstract] OR "Angola"[Title/Abstract] OR "Benin"[Title/Abstract] OR "Botswana"[Title/Abstract] OR "Burkina Faso"[Title/Abstract] OR "Burundi"[Title/Abstract] OR "Cape Verde"[Title/Abstract] OR "Cabo Verde"[Title/Abstract] OR "Cameroon"[Title/Abstract] OR "Central African Republic"[Title/Abstract] OR "Chad"[Title/Abstract] OR "Comoros"[Title/Abstract] OR "Democratic Republic of the Congo"[Title/Abstract] OR "Republic of Congo"[Title/Abstract] OR "Cote d'Ivoire"[Title/Abstract] OR "Ivory Coast"[Title/Abstract] OR "Djibouti"[Title/Abstract] OR "Egypt"[Title/Abstract] OR "Equatorial Guinea"[Title/Abstract] OR "Eritrea"[Title/Abstract] OR "Swaziland"[Title/Abstract] OR "Eswatini"[Title/Abstract] OR "Ethiopia"[Title/Abstract] OR "Gabon"[Title/Abstract] OR "Gambia"[Title/Abstract] OR "Ghana"[Title/Abstract] OR "Guinea"[Title/Abstract] OR "Guinea-Bissau"[Title/Abstract] OR "Kenya"[Title/Abstract] OR "Lesotho"[Title/Abstract] OR "Liberia"[Title/Abstract] OR "Libya"[Title/Abstract] OR "Madagascar"[Title/Abstract] OR "Malawi"[Title/Abstract] OR "Mali"[Title/Abstract] OR "Mauritania"[Title/Abstract] OR "Mauritius"[Title/Abstract] OR "Morocco"[Title/Abstract] OR "Mozambique"[Title/Abstract] OR "Namibia"[Title/Abstract] OR "Niger"[Title/Abstract] OR "Nigeria"[Title/Abstract] OR "Rwanda"[Title/Abstract] OR "Sao Tome and Principe"[Title/Abstract] OR "Senegal"[Title/Abstract] OR "Seychelles"[Title/Abstract] OR "Sierra Leone"[Title/Abstract] OR "Somalia"[Title/Abstract] OR "South Africa"[Title/Abstract] OR "South Sudan"[Title/Abstract] OR "Sudan"[Title/Abstract] OR "Tanzania"[Title/Abstract] OR "Togo"[Title/Abstract] OR "Tunisia"[Title/Abstract] OR "Uganda"[Title/Abstract] OR "Zambia"[Title/Abstract] OR "Zimbabwe"[Title/Abstract]) AND ("trial*"[Title/Abstract] OR "quasi experimental*"[Title/Abstract] OR "quasi*"[Title/Abstract] OR "quasi experimental*"[Title/Abstract] OR "non randomized"[Title/Abstract] OR "non randomised"[Title/Abstract] OR "experimental"[Title/Abstract] OR "before and after*"[Title/Abstract] OR "pre and post*"[Title/Abstract] OR "pretest and posttest"[Title/Abstract] OR "non equivalent"[Title/Abstract] OR "combination design*"[Title/Abstract] OR "implementation*"[Title/Abstract] OR "interrupted time series*"[Title/Abstract] OR "natural experiment*"[Title/Abstract] OR "pseudo experiment*"[Title/Abstract] OR "feasibility"[Title/Abstract])) AND (1987:2022[pdat])
