# Supplementary material for: A systematic review of behaviour change interventions to improve maternal health outcomes in sub-Saharan Africa
Source: PLOS Glob Public Health. 2024 Feb 20;4(2):e0002950. doi: 10.1371/journal.pgph.0002950 (PMC10878526; doi:10.1371/journal.pgph.0002950)
Supplement: Attachment — Submitted filename: Response to Reviewers.docx [file pgph.0002950.s007.docx]

Dr Francis Githae Muriithi,
Doctoral Research Fellow,
University of Birmingham

[fgm911@student.bham.ac.uk](mailto:fgm911@student.bham.ac.uk)

07/09/2023

Dear Academic Editor Dr Shifa S Habib, Reviewer 1 and 2,
PLOS Global Public Health

RE: Manuscript PGPH-D-23-00618– Response to journal requirements and reviewer comments

We are grateful for your consideration and feedback on our manuscript.

I resubmit our revised manuscript for your consideration on behalf of my co-authors.
I confirm that we have reviewed and provided an action/response to all reviewer comments. The specific actions/responses are detailed below this cover letter (on page 2 onwards) and row numbers refer to the Manuscript with tracked changes.

Journal requirement 1:	Page 2
Journal requirement 2:	Page 3
Response to reviewer 1:	Page 4 to 6
Response to reviewer 2:	Page 7 to 9

We hope you will find our response and changes to the manuscript appropriate.

Regards,

Dr Francis G. Muriithi
Corresponding Author

**Journal requirement 1**

We noticed you have some minor occurrence of overlapping text with the following previous publication(s), which needs to be addressed:

- <https://journals.plos.org/globalpublichealth/article?id=10.1371%2Fjournal.pgph.0000385>

In your revision ensure you cite all your sources (including your own works), and quote or rephrase any duplicated text outside the methods section. Further consideration is dependent on these concerns being addressed.

**Action/Response**

Thank you for your d**iligent** review of our manuscript and for providing your valuable suggestions. We have carefully considered each of your points and incorporated the suggested changes as outlined below.

We have run the manuscript on Turnitin software and identified a 2% similarity to the manuscript cited above.
We have made changes to the manuscript text in row 118 to 125,
Similarities in the methods section (row 227 to 242) are unrevised.
This is because the article screening and data extraction methods were similar and carried out by the same coauthors.
Similarities in the introduction part of the results section row 297 to 305 and the caption of figure 2 are due to similar writing style. These are unrevised.

Similarity in row 348 that signposts S1 Data is unrevised.

Similarities in the section between row 477 and 489 (Interpretation of findings)
We have used the word initiatives in place of efforts and avoidable in place of preventable.
See row 483 to 484. For uniformity, we have also replaced the word “preventable” with “avoidable” in the entire manuscript.

We have also restructured the sentence between row 488 and 490 and used alternative words of similar meaning where possible.

The title in row 507 has been changed from “Implications for clinicians, policymakers, and governments in African countries” to “Implications for maternal healthcare workers , policymakers, and governments in sub-Saharan Africa”

A more concise sentence is now in row 517 and 518 reading “……burden of avoidable maternal deaths in sub-Saharan Africa.”

The acknowledgement in row 542 – 544 is unchanged.

The other similarities to the paper are in the references – Specifically number 20 and 28. These are unchanged.

**Journal requirement 2**

Please review your reference list to ensure that it is complete and correct.

If you have cited papers that have been retracted, please include the rationale for doing so in the manuscript text or remove these references and replace them with relevant current references. Any changes to the reference list should be mentioned in the rebuttal letter that accompanies your revised manuscript. If you need to cite a retracted article, indicate the article’s retracted status in the References list and also include a citation and full reference for the retraction notice.

Action/Response

I confirm that We have scrutinised each of the 90 references in our manuscript and cross-checked their publication status on PubMed and journal websites. None are retracted.

**Response to reviewer comments: Reviewer 1**

Thank you for your diligent review of our manuscript and for providing your valuable suggestions. We have carefully considered each of your points and incorporated the suggested changes as outlined below.
 **Comment**

This is a very well written study, with a novel approach to collating behaviour change approaches to improving maternal health outcomes. The description of interventions shows the wide breadth of interventions being done across the continent, and how effective the interventions are. Many of the studies were published in the last 13 years. The fact that more interventions were directed at health seeking behaviour rather than healthcare worker behaviour and skills was also very interesting. Meaning there is greater drive towards primordial and primary prevention, over secondary and tertiary prevention. Would be interesting to know if the interventions were mostly designed by clinical or public health research groups.

**Action/Response**

Thank you for your kind comments on our work.

1. In the discussion I would perhaps state that this is the only systematic review 'according to your knowledge'

**Action/Response**
Thank you for this suggestion. We have revised this accordingly in row 433.

2. Did all the studies only measures effectiveness or were there other measures such as acceptability or outcomes. E.g., reduction in composite outcomes such as mortality or morbidity or haemorrhage or disease. In table 2 I would suggest adding a column on outcomes measured for completeness.
**Action/Response**
Thank you for this suggestion.
As per your suggestion, we have now added a detailed outcomes measures column on table 2. This information had been presented in supplementary file S1. data.

3. It would be interesting to know if you believe that behaviour interventions are scalable - and whether success in one regions means they could also be successful in other regions.

Action/Response

Thank you for this thought-provoking question. Indeed, the success of behaviour change interventions is very contextual.
To improve the manuscript, we have added this statement “Finally, although some behaviour change interventions were successful, it is important to consider other context specific factors that may influence external validity and scalability at other health facilities, regions or countries.” in the interpretation of findings section. See row 491 to 493.

We have also modified the recommendations for future research (See row 530 to 531) with the words “future research should assess the feasibility of solving local shared challenges in delivering maternal healthcare by shared learning within and between health facilities and identify factors that may influence the scalability of behaviour change interventions.”


4. Ultimately did the interventions have a reduction on the maternal mortality rate. If we don't know, this must be included as a limitation - as the overall framing of the paper implies that the impact of behaviour change is reduction in maternal mortality. However, the findings of the systematic review, overall suggest that behaviour change interventions are effective, without correlating change in behaviour with MMR.

Action/Response

Thank you for this suggestion. We agree with you and have added this as a limitation.
See row 508 to 511. “Finally, while we found evidence of effective behaviour change interventions, which could indirectly lead to a decrease in avoidable maternal deaths, a limitation is that none of these interventions directly correlated behaviour change with a reduction in the maternal mortality rate.”
Further we have added this statement in the suggestions for future research section “In future, behaviour change implementation research that is conducted at health facilities or regions with high maternal mortality should consider assessing the direct impact of the behaviour change intervention on maternal mortality rate. This may be a challenge to investigate at health facilities or regions where maternal deaths are a rare occurrence.”. See rows 535 to 539.

5. In the discussion it may be useful to include the concept of 'failure to rescue' as a reason to include more training interventions.

**Action/Response**
Thank you for this suggestion.
It has enabled us to view behavioural interventions in the context of a “system” instead of a solitary intervention.

Please see an addition in the discussion section row 471 to 478
“The finding of training as the dominant intervention, compared to other behaviour change interventions, can be partially elucidated by the "failure to rescue" concept. The concept describes a failure or delay in recognizing and responding patient deterioration leading to morbidity or mortality [90].

In an attempt to mitigate against failure to rescue, training of healthcare workers takes precedence over other elements in the healthcare system such as organisational, environmental, people, tasks, technology and tools factors that may equally impact healthcare delivery and outcomes [91, 92].”

Response to reviewer comments: Reviewer 2

Thank you for your diligent review of our manuscript and for providing your valuable suggestions. We have carefully considered each of your points and incorporated the suggested changes as outlined below.

Comments

Abstract:
I suggest that the authors add information on the methodology used for the systematic review. Include data source and eligibility criteria.

**Action/Response**
Thank you for this suggestion.
We had originally left the information out due to the word count limits set by the journal as the same information is available in the methodology section of the manuscript.
We have revised the abstract to add the data sources in – See rows 56 to 58. However, in the interest of the word count, we have left out the eligibility criteria as these are detailed in the methodology section of the manuscript.

Introduction:
In the first paragraph mention since when the decline in preventable maternal deaths has stagnated.

Action/Response

Thank you for pointing out this omission.
We have now revised the section see rows 85 to 96 to read as follows
“Following an average annual reduction rate (ARR) of 2.7% (UI 2.0% to 3.2%) from 2000 to 2015, the average global maternal mortality ratio (MMR) exhibited a stagnant trend between 2016 and 2020, at an ARR of -0.04% (UI -1.6% to 1.1%).The rate of decline in preventable maternal deaths has stagnated [1] A UN report estimates 287,000 global maternal deaths in 2020, resulting in an average global MMR of 223 per 100,000 live births (UI 202 to 255) with sub-Saharan Africa's MMR at 536 per 100,000 live births (UI 469 to 640), more than double the global average at an average global maternal mortality ratio (MMR) of 223 per 100,000 live births, with sub-Saharan Africa’s MMR being at 536 per 100,000 live births - more than twice the global average [1, 2]. . Moreover, in 2020 three sub-Saharan African countries exhibited MMRs surpassing 1000 deaths per 100,000 live births: South Sudan (1223; UI 746 to 2009), Chad (1063; UI 772 to 1586), and Nigeria (1047; UI 793 to 1565) [2].”

Regarding the most common causes of maternal death, insert the year to which the information is related to and the rates of each cause.

Action/Response

Thank you for this suggestion, we have revised this section adding the year and rates. See row 98 to 102.
It now reads as follows “Systematic review evidence from studies published between 2015 and 2020 reported that the most common causes of maternal death in sub-Saharan Africa are obstetric haemorrhage: 28.8% (95% CI=26.5%-31.2%), hypertensive disorders in pregnancy: 22.1% (95% CI=19.9%-24.2%), non-obstetric complications: 18.8% (95% CI=16.4%- 21.2%) and pregnancy-related infections: 11.5% (95% CI=9.8%-13.2%)”

I suggest that the authors structure the introduction as following:
Maternal Mortality rate
Main maternal cause of death
Interventions to reduce the MMR
Impact of behavioural interventions
Type of behavioural interventions and stakeholder’s influence.

Action/Response

Thank you for this suggestion which we have considered.
The subheadings were omitted in line with other PLOS GPH manuscripts.

Methods: review the exclusion criteria, there are not supposed to be the opposite of the inclusion criteria.
**Action/Response**
Thank you for this suggestion which is well noted.
We have now revised our wording in this section but very cautiously so as not to depart from the description in our protocol. See row 212 to 221.
It now reads as follows “Exclusion criteria were: 1) Articles reporting on studies with populations that neither accessed nor delivered maternal healthcare.2) Articles that described behaviour (i.e. measured or assessed patterns of current practice or behaviour) or influences on behaviour (e.g. qualitative, survey or observational studies) but did not intervene to try and change the behaviour; 3) Articles that did not include or report a behaviour change intervention; 4) Articles that described behaviour change interventions in countries that were not in sub-Saharan Africa and 5) Articles that were published before 1987 that is before the launch of the Safe Motherhood Initiative.”


Discussion: I suggest a detailed discussion on quality of data/studies included.

Action/Response

Thank you for the suggestion.
We presented the information regarding the quality of included studies in the initial part of the results section of the manuscript under “Inter-rater reliability and quality of included studies”
In addition and specifically within the manuscript, we have revised rows 457 to 470 to read as follows: “We found mixed-quality evidence from randomised trials and quasi-experimental studies conducted in 12 countries in sub-Saharan Africa countries namely Kenya, Ethiopia, South Africa, Malawi, Ghana, Nigeria, Egypt, Zambia, Uganda, Rwanda, Democratic Republic of Congo and United Republic of Tanzania. Using the Joanna Briggs tools to assess the quality of randomised trials and quasi-experimental studies we found that majority of the included studies had either a high (30 studies) or uncertain (11 studies) risk of bias. Only 10 studies had a low risk of bias. Scholars have reported in reviews that behavioural studies carry a high risk of bias, and it remains unclear whether this results from methodological flaws, reporting issues, or a mismatch of existing quality assessment tools to behavioural studies [85]. Tools such as the risk of bias justification table (RATIONALE) were designed to improve the quality of the conduct, reporting and assessment of behavioural trials [86]. It's crucial to consider our systematic review findings in light of the risk of bias categories we reported and the tool we utilised for quality assessment.”

Supporting information legends

We have updated the supporting information legends on page 52 of the manuscript with tracked changes (after the references).
They now read as follows:

**Supporting information**

Supplementary file **S1 Data**. Included studies and extracted data.
Supplementary file **S2 Data.** Framework analysis and coding.
Supplementary file **S3 Data.** ROBVIS generic dataset.
Supplementary file **S1 Table**. Search strategy and output.
Supplementary file **S2 Table**. Quality assessment.
Supplementary file **S3 Table.** PRISMA checklist.
