# Supplementary material for: A systematic review of behaviour change interventions to improve maternal health outcomes in sub-Saharan Africa
Source: PLOS Glob Public Health. 2024 Feb 20;4(2):e0002950. doi: 10.1371/journal.pgph.0002950 (PMC10878526; doi:10.1371/journal.pgph.0002950)
Supplement: Attachment — Submitted filename: Response to Reviewers.docx [file pgph.0002950.s008.docx]

Dr Francis Githae Muriithi,
Doctoral Research Fellow,
University of Birmingham

[fgm911@student.bham.ac.uk](mailto:fgm911@student.bham.ac.uk)

15/01/2024

Dear Academic Editor Dr Shifa S Habib, Reviewer 2 and 3,
PLOS Global Public Health

RE: Manuscript PGPH-D-23-00618R1 – Response to points raised by the editor, reviewers and journal requirements.

We are grateful for your consideration and feedback on our manuscript. I resubmit our revised manuscript for your consideration on behalf of my co-authors.

We have reviewed and provided an action/response to all journal requirements and reviewer comments. The specific actions/responses are detailed below this cover letter (on page 2 onwards). The row numbers refer to the Manuscript with tracked changes.

We have also reviewed our reference list and we confirm that none of the references is flagged up as retracted.

We hope you will find our response and changes to the manuscript appropriate.

Regards,

Dr Francis G. Muriithi
Corresponding Author

Journal requirement 1:

Editorial/Journal Comment:
Minor occurrence of overlapping text with my previous publication: <https://journals.plos.org/globalpublichealth/article?id=10.1371%2Fjournal.pgph.0000385>

Action/Response:
Thank you for sharing the concerns regarding some text overlapping with my previous publication.

We have run the manuscript through plagiarism software and identified the similarities. Some of the similarities are expected and not surprising. These are similarities such as authors and their affiliation details (rows 24 -39), acknowledgement and role of funding source statements (rows 638 - 648) and some references. This work is the output from the 2^nd^ workstream of a PhD project; hence, there are some similarities with the 1^st^ workstream.

I also confirm that my work had been cited appropriately, as in rows 113 – 114 and 131 -132 of the manuscript, with track changes. The cited reference (my first paper) is numbered 10 on the reference list.

Similar areas within the introduction, methods, and results have been reworded as illustrated in the manuscript with tracked changes to minimise the chances of similarity.

The specific parts of the manuscript we have reworded are:
In the Methods: Article processing (Rows 263 to 270), Data extraction (Row 273 to 277), In the Results: Figure 2 legend (Rows 339 to 344) and row 381. Similarities in the reference list have remained unaltered.

Journal requirement 2:

We noticed that you used "unpublished" in the manuscript. We do not allow these references, as the PLOS data access policy requires that all data be either published with the manuscript or made available in a publicly accessible database. Please amend the supplementary material to include the referenced data or remove the references.

Action/Response

Many thanks for your query about the term "Unpublished"

The word "unpublished" was in reference to other works that other researchers worldwide may have in their custody, e.g. grey literature, thesis etc

We had envisioned this as a weakness as far as comprehensiveness is concerned.

We have now reviewed it and decided that it does not add value to the limitations and therefore omitted it. We therefore do not need to amend the supplementary material.

Response to Reviewer 2:

Thank you for reviewing our work and your comments, which we found very useful.

**Comment:**
Please revise the exclusion criteria, those presented are the opposite of the inclusion criteria, this is not the definition of exclusion criteria. Please read about eligibility (inclusion and exclusion criteria), to know how to formulate them and edit accordingly.

**Action/Response:**
Thank you for your comment regarding the wording of our exclusion criteria.

I have read the Cochrane Handbook regarding the definition of eligibility criteria.

As a result, we have reworded the inclusion and exclusion criteria. See rows 191 to 233 in the manuscript with tracked changes.

**Response to Reviewer 3:**

Thank you for reviewing our manuscript. We very much appreciate your comments.

**Comment:**

Title: A Systematic Review of Behaviour Change Interventions to Improve Maternal Health Outcomes in Sub-Saharan Africa

**Action/Response:**

Thank you for suggesting that we reword the title of our manuscript from “Behavioural interventions for improving maternal health outcomes in sub-Saharan Africa: A systematic review” to the version above.

We have examined the title styles of similar works published by the PLOS GPH journal. We find that most articles have followed mainly a style similar to ours. However, considering your suggestion, we accepted it as the new format may appeal better to our intended readership. In addition, it emphasises the word “change,” which was missing from the initial title.

The change in the title is reflected in rows 1 and 2 of the manuscript with tracked changes.

**Comment:**

**Strengths:**

Innovative Frameworks: The utilization of the AACTT framework and BCW adds depth and clarity to the review, providing a solid foundation for categorizing behaviours and intervention strategies.

Comprehensive Scope: The review covers a wide range of studies from various Sub-Saharan African countries, offering a comprehensive overview of the behavioural landscape related to maternal health.

**Limitations:**

Risk of Bias: The majority of included studies exhibited a high or uncertain risk of bias, potentially impacting the reliability of findings. The review acknowledges this limitation but does not delve deeply into its implications.

Outcome Measurement: While effective interventions are identified, the review falls short of directly correlating behaviour change with a reduction in the maternal mortality rate. Future research might benefit from exploring this link more explicitly.

**Action/Response:**

We are grateful for your compliments and suggestions regarding the strengths and limitations of our work.

We have revised the strengths and limitations to reflect your suggestions. See rows 549 to 572

In addition, we have revised the suggestions for future research in line with the limitations we identified in the systematic review and your very useful tips. See rows 594 to 620.
